# A Synthetic, Xeno-Free Peptide Surface for Expansion and Directed Differentiation of Human Induced Pluripotent Stem Cells

**DOI:** 10.1371/journal.pone.0050880

**Published:** 2012-11-30

**Authors:** Sha Jin, Huantong Yao, Jennifer L. Weber, Zara K. Melkoumian, Kaiming Ye

**Affiliations:** 1 Department of Biomedical Engineering, College of Engineering, University of Arkansas, Fayetteville, Arkansas, United States of America; 2 Corning Incorporated, Life Sciences, Corning, New York, United States of America; University of Rochester, United States of America

## Abstract

Human induced pluripotent stem cells have the potential to become an unlimited cell source for cell replacement therapy. The realization of this potential, however, depends on the availability of culture methods that are robust, scalable, and use chemically defined materials. Despite significant advances in hiPSC technologies, the expansion of hiPSCs relies upon the use of animal-derived extracellular matrix extracts, such as Matrigel, which raises safety concerns over the use of these products. In this work, we investigated the feasibility of expanding and differentiating hiPSCs on a chemically defined, xeno-free synthetic peptide substrate, i.e. Corning Synthemax® Surface. We demonstrated that the Synthemax Surface supports the attachment, spreading, and proliferation of hiPSCs, as well as hiPSCs’ lineage-specific differentiation. hiPSCs colonies grown on Synthemax Surfaces exhibit less spread and more compact morphology compared to cells grown on Matrigel™. The cytoskeleton characterization of hiPSCs grown on the Synthemax Surface revealed formation of denser actin filaments in the cell-cell interface. The down-regulation of vinculin and up-regulation of zyxin expression were also observed in hiPSCs grown on the Synthemax Surface. Further examination of cell-ECM interaction revealed that hiPSCs grown on the Synthemax Surface primarily utilize α_v_β_5_ integrins to mediate attachment to the substrate, whereas multiple integrins are involved in cell attachment to Matrigel. Finally, hiPSCs can be maintained undifferentiated on the Synthemax Surface for more than ten passages. These studies provide a novel approach for expansion of hiPSCs using synthetic peptide engineered surface as a substrate to avoid a potential risk of contamination and lot-to-lot variability with animal derived materials.

## Introduction

Unlike human embryonic stem cells (hESCs), human induced pluripotent stem cells (hiPSCs) can be derived from the patients’ own cells. This raises hopes for generating patient-specific cells to treat many otherwise incurable diseases through cell replacement therapy [1], [2], [3]. Although the clinical application of hiPSCs is still at its early stage, the safety of these cell products has already received broad attention. For clinical use of these cells, it is highly desirable to have culture methods that are scalable and use chemically defined raw materials for both cell expansion and differentiation. The development of serum-free embryonic stem cell culture media, such as mTeSR, provides a chemically defined cell culture medium for expansion of hiPSCs [4], [5]. However, development of chemically-defined culture surfaces to support hiPSCs growth and differentiation remains elusive.

Currently, hiPSCs are maintained and differentiated on either feeder layer cells [6] or Matrigel coated cell culture dishes [7], [8], [9], [10]. Compared to feeder layer cells, the preparation of Matrigel coated surfaces is a relatively easy and inexpensive process. However, Matrigel is an undefined mixture of extracellular matrix (ECM) proteins extracted from the Engelbreth-Holm-Swarm (EHS) mouse sarcoma. It consists of laminin, collagen IV, heparan sulfate proteoglycans, entactin, nidogen, and some undefined factors. As a result, the quality and composition of Matrigel varies from lot to lot. There is also a risk of potential contamination with animal-derived viruses, raising significant safety concerns over Matrigel use in clinical applications [11]. Hence, there is a great need for the development of xeno-free, synthetic surfaces that capable of providing necessary stem cell niches to allow hiPSCs expansion and differentiation in a serum-free defined media.

It has been well documented that the in vitro expansion of stem cells relies on cell-ECM interaction, which occurs between cell surface adhesion molecules, such as integrins and their counterparts in ECM. This interaction enables cells to attach, spread, proliferate, migrate, and differentiate on a substrate. Accordingly, a chemically defined synthetic substrate can be developed by coating or functionalizing the substrate with chemically synthesized materials that mimic the ligands of cell surface adhesive molecules. Many examples of this approach are available in literatures. For example, the coating of a substrate with single or multiple ECM proteins has been explored for hESC maintenance [12], [13], [14]. While the coating of a substrate with one or two ECM proteins has been successful, it is not ideal because the production of recombinant ECM proteins is still very expensive. However, encouraged by these successes, the use of a motif rather than a full protein to support hiPSC growth and differentiation has been proposed and rigorously examined. A line of evidence reveals that the cell surface adhesive molecule binding motifs of ECM proteins play an imperative role in guiding cell attachment and spreading on a substrate coated with the ECM protein [15]. These motifs also transmit physiochemical signals to a cell thereby altering the cell’s fate [16]. Thus, it is conceivable that surfaces displaying cell adhesion promoting motifs can support hiPSC attachment and growth by recapitulating integrin matrix engagement found in cell-ECM interaction. Most of these cell adhesion motifs are short peptides that can be chemically synthesized using xeno-free raw materials. For example, the use of small peptides to mimic the function of laminin, one of the critical ECM proteins, has been widely explored [17], [18], [19]. Laminin has at least 15 isoforms, each consisting of α, β, and γ chains [20]. The cell binding domains of laminin isoform 1 (laminin-1) has been characterized using fibroblasts adhesion model. As a result, four peptide sequences located on different laminin chains have been identified [16]. It has been shown by an integrin blocking assay that treatment of hES/iPS cells with anti-integrin α_5_β_1_, α_v_β_3_, or α_v_β_5_ antibodies can suppress hESC attachment to the substrates coated with these peptides [15], [21]. Despite the success in creating these synthetic substrates for hESC expansion and differentiation, the requirement of using either conditioned medium, which is not chemically defined [22], [23], [24], or an inhibitor of rho-associated kinase (ROCK), such as Y27632 [25], [26], complicates the process. The use of ROCK inhibitor is not desirable for clinical applications. It has been shown that inhibiting ROCK could lead to kidney failure as well as acute hypotension caused by the relaxation of the vascular smooth muscles [27], [28]. To overcome these issues, the Synthemax Surface has been recently developed by Corning Inc. It is based on the Arg-Gly-Asp (RGD) motif containing a short peptide, which is derived from the vitronectin protein and immobilized on the acrylate coating. The Synthemax Surface was shown to successfully support hESC growth and differentiation in chemically defined media [11], [29]. This example proves that a short peptide can indeed recapitulate integrin ECM engagement found in hESCs grown on Matrigel-coated substrate.

**Figure 1:**
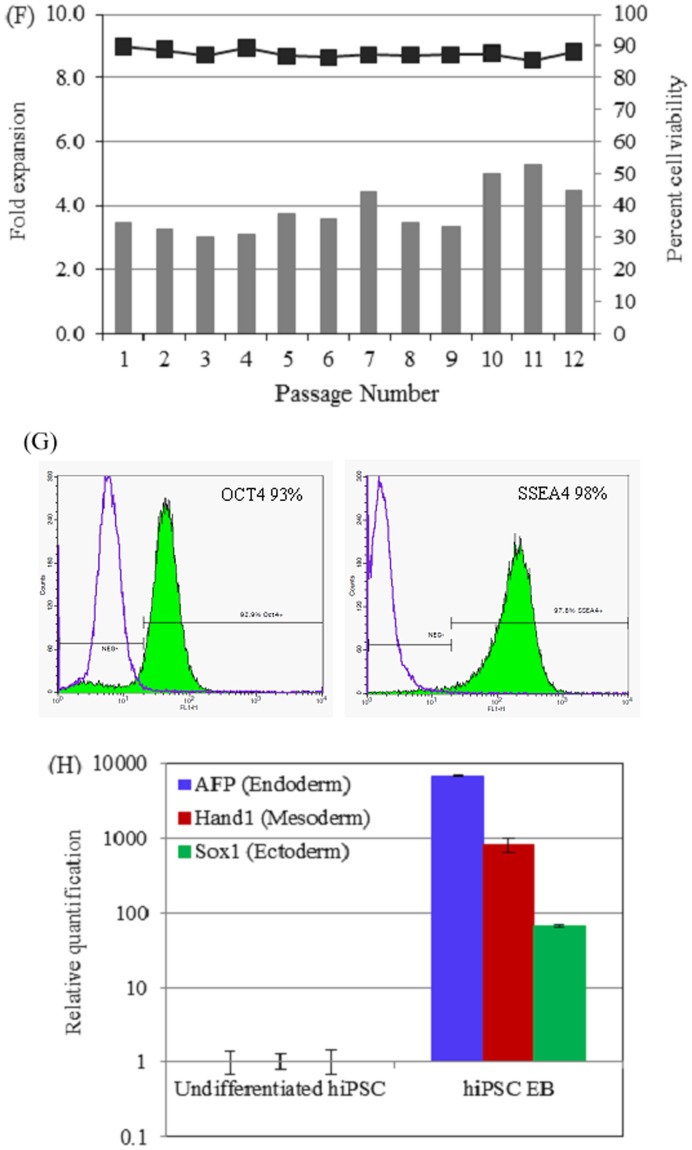
Comparison of hiPSC proliferation on Synthemax Surface and Matrigel. Brightfiled image of hiPSC colony formed on SM (A) and MG (B) surface. Scale bar: 50 µm. (C) Cell proliferation on different surfaces determined by counting viable cell number every 24 h. Data is presented as mean ± SD from three independent experiments. p-value: 0.7008 (D) Detection of pluripotency of hiPSCs on Synthemax Surface by quantitative real-time PCR analysis. The expression level of pluripotent marker gene in cells maintained on Matrigel-coated surface was detected for comparison. (E) Immunofluorescent staining of stem cell pluripotency markers, OCT4 and SSEA4 in hiPSCs maintained on SM surface for ten passages. Scale bar: 100 µm. MG, Matrigel; SM, Synthemax Surface. (F) Cell expansion and viability in the course of twelve consecutive passages on Synthemax Surface. (G) Flow cytometry analysis of OCT4 and SSEA4 markers after ten passages of hiPSCs on Synthemax Surface. (H) qPCR analysis of germ layer markers after induced differentiation of hiPSC maintained on Synthemax Surface for more than ten passages.

The goal of this study was to determine whether hiPSCs can be maintained over a long period of time and differentiated on the Synthemax Surface. A line of evidence suggests that hiPSCs and hESCs exhibit some differences, despite similar patterns in global transcriptome assessment [30], [31]. It has been found that a subset of 318 genes differentially expressed between these two types of cells [31]. This small set of genes may represent a genetic memory of the ancestor cells from which the hiPSCs were derived [30]. Thus, it is critical to assess whether the Synthemax Surface is suitable for hiPSC maintenance and differentiation. The underlying mechanisms of these processes have also been characterized in this study.

**Figure 2:**
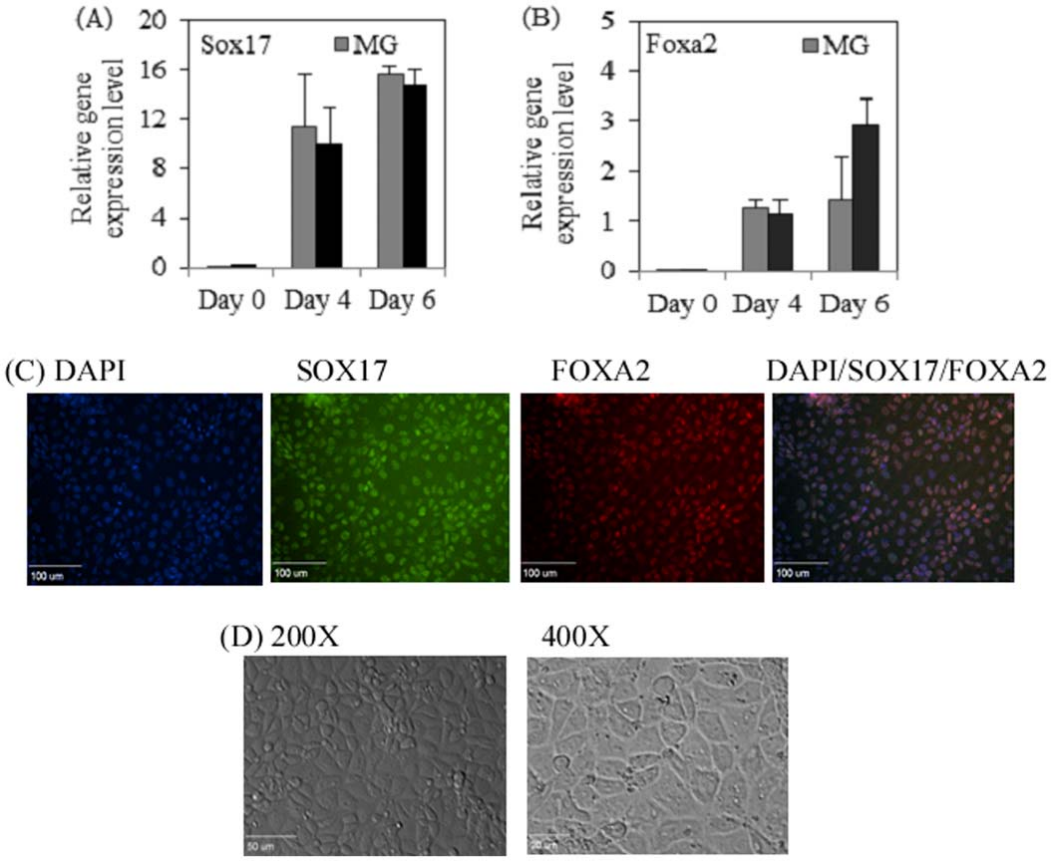
Definitive endoderm (DE) differentiation of hiPSC on Matrigel and Synthemax Surface. The expression of SOX17 (A) and FOXA2 (B) was normalized to their expression levels in adult pancreatic (AP) tissues. Data is presented as mean ± SD from three independent experiments. p-value = 0.917 for SOX17 and p-value = 0.668 for FOXA2. (C) Immunofluoresent staining for SOX17 and FOXA2 DE markers in hiPSCs differentiated on SM. (D) Phase contrast images of hiPSC morphology after DE differentiation on SM. Majority of the cells exhibited endothelial-like morphology on day 6 post differentiation.

## Materials and Methods

### 2.1. hiPSC Expansion

The hiPSC line IMR90 and Gibco® Episomal hiPSC Line were acquired from the WiCell Institute and Invitrogen, respectively. IMR90 cells were maintained on Matrigel (BD Biosciences, CA) coated polystyrene tissue culture treated dishes in a chemically defined medium mTeSR1 (Stem Cell Technologies, Vancouver, Canada) at 37°C and 5% CO_2_ with daily medium exchange. The growth factor reduced Matrigel (BD Biosciences) was diluted 1∶100 in a DMEM/F12 medium (HyClone, Utah) and coated to cell culture dishes for one hour. hiPSCs were seeded and cultured on Matrigel-coated dishes in the undifferentiated state. Similarly, Gibco hiPSC were maintained on Geltrex™ (Gibco) coated polystyrene tissue culture treated dishes in mTeSR1 prior to expansion on Synthemax® surface (Corning, NY). Subculture of hiPSCs was performed by treating these cells with 1 mg/mL dispase (Stem Cell Technologies), followed by a gentle scraping of cell colonies from the culture dishes with a split ratio of 1∶3 to 1∶5 every three or four days. hiPSCs were also seeded onto a synthetic peptide functionalized Synthemax Surface (Corning, NY) at a density of 50,000 cells/cm^2^ in the mTeSR1 medium. To determine hiPSC expansion on these surfaces, cells were harvested with 0.05% trypsin-EDTA for 5 min, and the total cell number, as well as the viable cell number, was counted using a Cell Counter (Bio-Rad) following trypan blue staining. Cell morphology was monitored daily to insure that the cells maintained an undifferentiated state. To monitor genomic integrity, cell samples for all conditions were submitted for karyotype analysis by G-banding (WiCell Research Institute Cytogenetics Lab).

**Figure 3:**
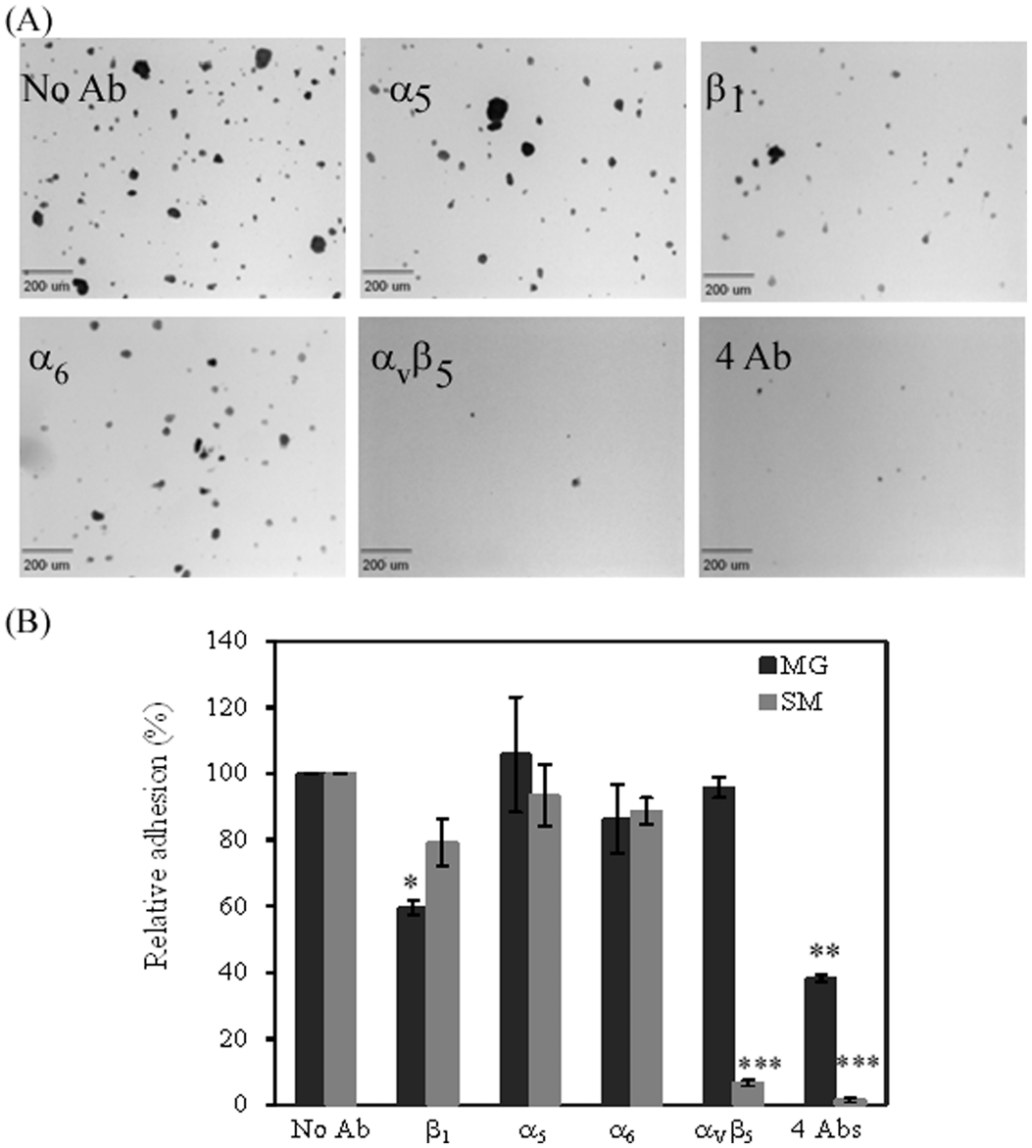
The role of integrin receptors in hiPSC adhesion to Synthemax Surface and Matrigel. hiPSCs were incubated with indicated anti-integrin antibodies before seeding onto the corresponding surface. (A) Micrographic images of cell attachment to the Synthemax Surface after integrin treatment. Scale bar: 200 µm. (B) Relative cell adhesion determined at 1 h post seeding. A cell adhesion was estimated by counting the number of colonies in 14 randomly selected fields. The relative cell adhesion was normalized to that for integrin-untreated cells. All results were expressed as the mean ± SD (n = 14) in two typical measurements of more than 3 independent experiments. *: p = 0.0093; **: p = 0.0002; ***: p<2.75E-10. Symbols: Ab, antibody; MG, Matrigel; SM, Synthemax Surface.

### 2.2. Induction of hiPSC Differentiation

To induce definitive endoderm (DE) differentiation, undifferentiated hiPSCs were seeded onto Matrigel and Synthemax Surfaces and cultured overnight in the mTeSR medium after dispase treatment (1 mg/mL) and gentle scraping. Cells were then fed with a definitive endoderm (DE) induction medium containing RPMI1640, B27 (Invitrogen), 1 mM sodium-butyrate, and 4 nM activin A. The sodium butyrate concentration was reduced to 0.5 mM after 24 h differentiation. The differentiation medium was changed every other day until day 7 post differentiation induction. To induce embryoid body (EB) formation, hiPSC colonies were harvested after thirteen passages on Synthemax Surface, and seeded onto ultra-low attachment six-well plates for EB formation assay. Cells were maintained in Iscove’s Modified Dulbecco’s Medium (IMDM) supplemented with 10% FBS and re-fed every 2 days for 14 days. EBs were frozen in CryoStor-10 cryopreservation medium. qRT-PCR was performed on EBs to confirm the presence of the three germ layer markers.

**Figure 4:**
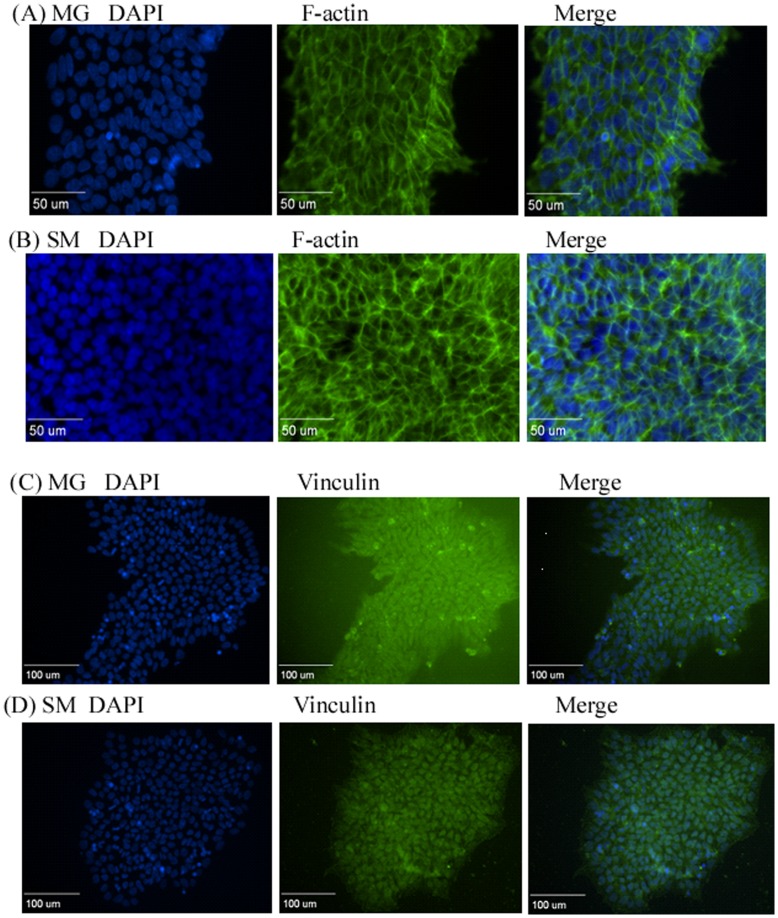
Organization of cytoskeletal structure proteins in hiPSCs grown on different surfaces. (A, C, E): MG surface; (B, D, F): SM surface**.** hiPSCs were fixed after growing on the corresponding surfaces for two days and stained with antibodies against F-actin, vinculin, and zyxin, respectively. Arrows indicate denser and more pronounced actin filament expression. Scale bars: (A–B, E–F) 50 µm, (C–D) 100 µm. MG, Matrigel; SM, Synthemax Surface.

### 2.3. Immunofluorescence Microscopy

To examine the protein expression in hiPSCs, immunofluorescent staining was performed as described in our previous work [32]. Mouse anti-human octamer-binding transcription factor 4 (OCT4) (1∶100), Stage-specific embryonic antigen-4 (SSEA4) (1∶100) (Millipore, Billerica, MA), SOX17 (1∶50; R&D Systems); rabbit anti-human FOXA2 (1∶1000; Abcam), vinculin (1∶50; Santa Cruz, CA), zyxin (1∶100; Sigma), and Alexa Fluor 488 phalloidin (5 U/ml, Invitrogen) were used as primary antibodies. The goat anti-mouse Alexa Fluor 488 IgG_1_ (1∶200), goat anti-mouse Alexa Fluor 488 IgG_3_ (1∶200) (Life Technologies, Carlsbad, CA), goat anti-mouse IgG FITC (1∶100; Sigma), donkey anti-rabbit IgG TRITC (1∶50) (Jackson Immuno research Laboratories In.), and mouse anti-rabbit IgG FITC antibodies (1∶150; Sigma) were used as the secondary antibodies. Cells were labeled with DAPI (4′,6-diamidino-2-phenylindole) nuclear stain. Four drops of DAPI (Vector Laboratories, Inc. Burlingame, CA) were added to the each well and incubated for one minute. The specificities of these antibodies were verified against their corresponding isotype controls. With the Slidebook imaging software, we first optimized the exposure time and fluorescence sensitivity by using our negative control samples, to ensure no auto-fluorescence or false positive imaging. The same parameters were then used for imaging the rest of the samples.

**Figure 5:**
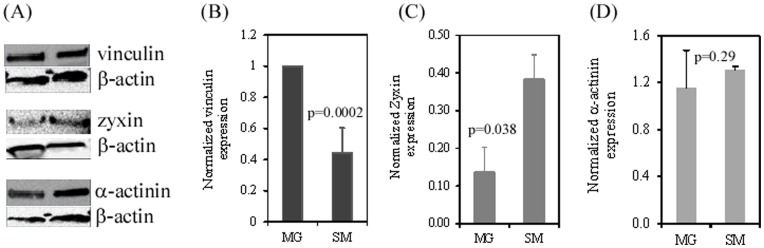
Cytoskeletal protein expression in hiPSCs grown on Matrigel and Synthemax surface. Cells were harvested at 48 h post seeding and total proteins were extracted for Western blot analysis. At least three independent experiments were performed and data were presented as mean ± SD. (A) Vinculin, zyxin, and α-actinin expression detected by Western blot analysis. (B-D) Relative protein expression using β-actin as a loading control. Semi-quantification of protein expression was performed by Kodak 1D gel imaging software. MG, Matrigel; SM, Synthemax Surface.

### 2.4. Adhesion-blocking Assay

Adhesion-blocking assays were carried out as described previously with some modifications [13]. In brief, approximately 0.5×10^6^ hiPSCs were incubated in a phenol red-free CMRL 1066 medium (Cellgro) containing 0.35% bovine serum albumin (BSA) at 37°C for 30 min in the absence or presence of mouse anti-human integrin antibodies (10 µg/ml, Chemicon International, Billerica, MA). After incubation, cells were seeded onto either Synthemax Surface (SM) or Matrigel (MG) coated 6-well plates. The cells were allowed to adhere to the surfaces at 37°C in a 5% CO_2_ incubator for 1 h. Nonattached cells were carefully removed by washing the wells three times with CMRL/0.35% BSA and once with PBS. The attached cells were fixed with 100% ethanol at room temperature for 5 min and stained with 0.4% crystal violet in methanol for 5 min, followed by a thorough wash with MilliQ water. Cell attachment was visually scored by counting the number of cell colonies in seven randomly selected fields. The average of these numbers was normalized to a non-blocking control and used as an index for quantifying cell attachment. The microscopy images were captured using an inverted Olympus IX71 microscope (Tokyo, Japan) equipped with a cooled charge-coupled device (CCD) camera (Qimaging Retiga 4200, Surrey, Canada) controlled by a Lambda 10-3 (Sutter Instrument Company, CA).

### 2.5. Quantitative Real-time PCR (qRT-PCR)

TaqMan quantitative real-time (qRT-PCR) analysis was performed according to our previous work [32], [33]. Briefly, total RNA was extracted from cells using an RNeasy kit (Qiagen, Valencia, CA). The genomic DNAs were eliminated from RNA samples during extraction. After quantifying with a Synergy MX microplate reader (BioTek, Winooski, VT), qRT-PCR was performed to detect pluripotent stem cell (OCT4) and differentiation marker genes with TaqMan Master Mix from Applied Biosystems, as described elsewhere [32]. The following primer-probe pairs were used for mRNA detection. OCT4: Forward 5′- GAAACCCACACTGCAGCA -3′, Reverse 5′-CACATCCTTCTCGAGCCCA-3′, and probe 5′ FAM -CAGCCACATCGCCCAGCA - BHQ1 3′. The following TaqMan® Assay IDs were used to detect all three germ layer markers: AFP: Hs00173490_m1; Hand1: Hs02330376_s1; SOX1: Hs01057642_s1 (Invitrogen). The RNA samples that were not reversely transcribed were used as controls in order to rule out any possibilities of genomic DNA contamination in the qRT-PCR assay.

### 2.6. Western Blot

To characterize cytoskeletal protein expression, total cell proteins were extracted using a cell lysis buffer containing 50 mM Tris-HCl (pH 7.4), 1% Triton X-100, 0.1% SDS, 150 mM NaCl, 1 mM Dithiothreitol, and 1 mM phenylmethylsulfonyl fluoride. Western blot was carried out as described in our previous work [34], [35]. Rabbit anti-human α-actinin (1∶1000; Sigma), anti-human zyxin (1∶1000; Sigma), and anti-vinculin antibodies (1∶200; Santa Cruz) were used as primary antibodies, whereas goat anti-rabbit IgG horseradish peroxidase (HRP) conjugates were used (1∶1000; Sigma) as secondary antibodies. β-actin served as a control for semi-quantitative analysis of Western blot. It was detected using mouse HRP-conjugated β-actin antibodies (1∶35,000; Sigma).

### 2.7. Statistical Analyses

Data were presented as mean ± standard deviation. The statistical analysis was performed based on ANOVA and post-hoc significant difference test. Statistical significance was determined at *p*0.05.

## Results

### 3.1. Attachment, Spreading, and Proliferation of hiPSCs on Chemically Defined Synthetic Peptide Surface

First, we examined whether hiPSCs can attach, spread, and proliferate on the Synthemax Surface. hiPSCs were seeded onto both Synthemax and Matrigel surfaces and cultured in a chemically defined mTeSR1 medium. The attachment of hiPSCs to the surfaces was observed and imaged using an inverted phase contrast microscope, whereas cell proliferation was determined by counting viable cells every 24 hours until day 4. The observation of cell colony morphology on these surfaces revealed that cell colonies on the Synthemax Surface became more compact than those on Matrigel (Figs. 1A and B). This was supported by counting the number of viable cells per cm^2^ for both surfaces. As shown in Fig. 1C, a higher cell number was observed on Synthemax Surface. The cell doubling time on Synthemax Surface was 41.2 hours, compared to 43.6 hours on Matrigel. ANOVA statistical test shows p-value of 0.7008, indicating there is no significant difference between doubling time for cells cultured on Matrigel and Synthemax Surface.

Next, we determined whether hiPSCs can be maintained in the undifferentiated state on the Synthemax Surface over the course of multiple passages. OCT4 mRNA expression in cells cultured on Synthemax Surface after one passage and ten passages were analyzed and compared with the expression level in cells cultured on Matrigel-coated surface (Fig. 1D). The OCT4 mRNA expression was similar for cells on both surfaces. We did not observe significant difference among the samples tested (p-value = 0.5190). In addition, immunofluorescent microscopy was performed to monitor the expression of embryonic stem cell pluripotency markers: OCT4 and stage-specific embryonic antigen 4 (SSEA4) in hiPSCs maintained on Synthemax Surface. As presented in Fig. 1E, hiPSCs sustained a high level of expression of OCT4 and SSEA4 after ten passages, suggesting their pluripotent status. The same results were demonstrated with the second hiPSC line. As show in Figure 1F, hiPSC were successfully maintained on Synthemax Surface in mTeSR1 medium for more than ten passages with the stable fold expansion and high viability. After ten passages on Synthemax Surface, hiPSC maintained high level of pluripotency markers, OCT4 and SSEA4 (Figure 1G) and ability to differentiate into all three germ layers (Figure 1H). Together, these data demonstrate that hiPSCs can self-renew on Synthemax Surface for more than ten passages without losing their immunophenotype and pluripotency.

### 3.2. Directed Differentiation of hiPSC on Synthetic Peptide Surface

We next investigated whether hiPSCs retain their ability to differentiate into a specific lineage, such as a DE lineage. The differentiation of DE lineage is the first and also the most critical step in hESC pancreatic differentiation [36], [37], [38]. Thus, demonstration of hiPSC directed DE differentiation on peptide surface could help develop a xeno-free differentiation system to generate transplantable β-cells from hiPSCs for diabetes therapy. To perform DE differentiation, the hiPSCs were cultured on the Synthemax Surface followed by induction of DE differentiation using special medium described in Materials and Methods section. The expressions of DE markers SOX17 and FOXA2 in differentiated hiPSCs were detected by qRT-PCR. As presented in Fig. 2, similar expression levels of the marker genes were observed in cells differentiated on either Synthemax or Matrigel surfaces since statistical test indicated p-value of 0.917 and 0.668, respectively, for SOX17 and FOXA2. The qRT-PCR results are supported by immunofluorescent microscopy analysis (Fig. 2E). The expressions of DE marker proteins for SOX17 and FOXA2 were observed in hiPSCs differentiated on the Synthemax Surface (Fig. 2C). The DE morphology was observed on day 5 post induction of differentiation (Fig. 2D). Our results indicate that the Synthemax Surface provides the appropriate niche environment that supports both the expansion and the directed differentiation of hiPSCs.

### 3.3. Integrin α_V_β_5_ Plays an Essential Role in hiPSCs Adhesion to the Synthetic Peptide Surface

The success in expanding and differentiating hiPSCs on the synthetic peptide surface posed a fundamental question about the molecular mechanisms of cell attachment to this surface. We hypothesized that hiPSCs attach to the synthetic peptide substrate through integrin engagement. To identify which integrins are involved in mediating hiPSC attachment to the Synthemax Surface, we performed the adhesion inhibition assay by blocking various integrins with anti-integrin antibodies before seeding the cells to the Synthemax Surface. Cells seeded on the Matrigel substrates served as control for this assay. We found that the blocking of integrin α_V_β_5_ significantly inhibited the attachment of hiPSCs to the Synthemax Surface by 93% (Fig. 3A). On the other hand, no significant reduction in cell adhesion by α_V_β_5_ antibody was observed for hiPSCs on Matrigel surface (Fig. 3B). The blocking of integrins α_5_, α_6,_ and β_1_ only reduced the cell adhesion to the Synthemax Surface by 20, 6, and 11%, respectively. Cells treated with all four antibodies against integrins α_5_, α_6_, β_1_, and α_V_β_5_ completely abolished the attachment of hiPSCs to the Synthemax Surface. These results suggest that hiPSCs interact with the Synthemax Surface mainly through the integrin α_V_β_5_. Our results are consistent with previous studies that demonstrated recognition of recombinant vitronectin protein by integrin α_V_β_5_ [13], [39] since the Synthemax Surface contains peptide sequence derived from vitronectin proteins.

In contrast, the blocking of integrin β_1_ led to 40% reduction in hiPSC adhesion to the Matrigel surface, indicating that integrin β_1_ plays an important role in hiPSC attachment to Matrigel. The results are similar to the observation reported previously, which found that integrin β_1_ is required for hiPSCs adhesion and proliferation on Matrigel-coated surfaces [39]. The blocking of integrin α_6_ resulted in a 14% reduction in hiPSC adhesion to Matrigel. The combination of antibodies against integrins α_5_, α_6_, β_1_, and α_V_β_5_ resulted in a 62% reduction of cell adhesion to the Matrigel surface. These results suggest that multiple integrins are involved in mediating hiPSCs adhesion to the Matrigel surface.

### 3.4. Organization of Cytoskeleton Structures of hiPSCs Grown on the Synthemax Surface

To investigate physicochemical cues provided by the Synthemax Surface for hiPSC proliferation and differentiation, we examined the organization of the cytoskeleton structures, such as actin filaments (F-actin) and vinculin during hiPSC growth on this surface. Actin filaments are the smallest filamentous proteins involved in both cell structure (a static role) and cell movement (a dynamic role). They are the main components of the cytoskeleton. Vinculin is another cytoskeletal protein that is recruited from the cytoplasm to the focal adhesions. Its recruitment is regulated by either external, internal, or both signals [40]. This recruitment is critical for cell spreading and migration [41], [42]. To investigate actin polymerization in hiPSCs grown on the Synthemax Surface, cells were fixed, permeabilized, and labeled with phalloidin after 48 hours in culture. As shown in Fig. 4, hiPSCs grown on the Synthemax Surface exhibited the actin filament network that was significantly different from that formed in cells grown on the Matrigel surface. While cells grown on both surfaces showed actin stress fibers at focal adhesions, the accumulation of denser and broader actin filaments between the cell-cell interfaces occurred only in cells grown on the Synthemax Surface. This may suggest the enhanced actin filament networks in hiPSCs when they are grown on the Synthemax Surface. Vinculin staining revealed its comparable distribution in hiPSCs grown on both Synthemax and Matrigel surfaces (Fig. 4C & D). However, cells grown on the Synthemax Surface expressed less vinculin, as revealed by a Western blot analysis (Fig. 5).

In addition, we found that there is a higher expression of zyxin protein in these cells. Zyxin is a zinc-binding phosphoprotein that concentrates at focal adhesions and along the actin cytoskeleton. The immunofluorescent microscopy showed that hiPSCs expressed and formed polygonal structures on both Synthemax and Matrigel surfaces (Fig. 4E). However, a semi-quantitative Western blot assay revealed a significant up-regulation of zyxin in cells grown on the Synthemax Surface (Fig. 5). Because zyxin is directly involved in cell spreading and proliferation and is inversely correlated to differentiation [43], its up-regulation may contribute to enhanced cell attachment and proliferation on the Synthemax Surface, as described above. Finally, α-actinin expressions in cells grown on the Synthemax and Matrigel surfaces were comparable (Fig. 5D).

## Discussion

*In vivo*, stem cells interact with various microenvironments, including tissue niches that regulate gene expressions, thus affecting cell fate. These niches can be categorized into three groups. The first group is signaling molecules, such as transforming factor-β (TGF-β) superfamily members and fibroblast growth factors (FGFs). These signaling molecules regulate stem cell self-renewal by activating Smad2/3 signaling and suppressing BMP (bone morphogenetic protein) signaling [44], [45], [46], [47], [48], [49]. The second group is extracellular structures that support cell attachment, interaction, and migration. These local cellular microenvironments act on stem cells in an indirect fashion mediated by cell-ECM and/or cell-cell contacts [50]. The third group is physicochemical conditions to which stem cells are exposed. These physicochemical factors include pH, ionic strength, oxygen level of a cell culture medium, and the mechanical/chemical properties and topographic features of a substrate that supports cell growth. Cells also generate their own mechanical signals through cell traction actions. These actions are driven by forces generated from different molecular interactions that are primarily mediated by the motor protein myosin II and other cytoskeletal systems, such as microtubules [51], [52], [53], [54], [55].

Therefore, for optimal expansion and differentiation of stem cells for clinical applications, it is critical to design a cell culture environment that mimics cell niches using defined xeno-free materials. In this work, we showed that the synthetic, xeno-free peptide surface, Synthemax Surface, supports long-term maintenance and directed differentiation of hiPSCs in a defined medium. This will offer a better solution to produce clinical-grade hiPSCs and their derivatives for therapeutic applications. We observed that hiPSC colonies formed on a peptide surface exhibit more compact morphology than those on Matrigel surface. The characterization of cell-ECM interaction in hiPSCs grown on the Synthemax Surface revealed that the integrin α_V_β_5_ plays a key role in mediating the interaction between hiPSCs and the Synthemax Surface, which is functionalized with synthetic RGD-containing peptide sequence from vitronectin protein. In contrast, we found that cells grown on Matrigel coated surface utilize multiple integrins for mediating cell-ECM interaction. These results are consistent with other reports in the literature [15]. Vuoristo et al showed that blocking integrin β_1_ subunit can completely obstruct the adhesion of hESCs to a basement membrane protein coated substrate [56]. Meng and co-workers found that the integrin α_6_, β_1_, α_2_β_1_ and α_v_β_3_ regulate hESC attachment to Matrigel coated substrates in a chemically defined medium [15]. In summary, our results demonstrated that the engagement of a single α_v_β_5_ integrin on the cell surface was sufficient to maintain the self-renewal of hiPSCs on the chemically-defined animal-free Synthemax Surface.

Cytoskeleton is a mechanotransduction component that is related to integrin signaling transduction pathways. The cytoplasmic domains of integrins bind to the cytoskeleton through adapter proteins like vinculin, α-actinin, and phosphorylated-focal adhesion kinase (p-FAK). These protein complexes form focal adhesions, i.e., sub-cellular sites that receive and transduce physiochemical signals from a substrate [57], [58] to influence cell morphology [59], [60], adhesion [61], proliferation [62], motility [63], differentiation [64], and cell fate [55]. The cytoplasmic domains of β-integrin also interact with talin, filamin, tesin, and other focal adhesion proteins to stabilize or destabilize focal adhesions [55], [65], [66], [67], leading to the remodeling of microfilament and microtubule networks, which in turn affects gene transfer and expression. We observed similar level of α-actinin expression on both the Synthemax and the Matrigel surfaces, but found down-regulation of vinculin and up-regulation of zyxin in hiPSCs grown on the Synthemax Surface (Fig. 5). While the mechanism of these changes in cell cytoskeletal proteins is unclear, it may still indicate a reorganization of cellular molecules and focal adhesions, which facilitates the spreading and self-renewal of hiPSCs on substrates, such as peptide surface used in this work.

Another important finding of this study was that a synthetic Synthemax Surface not only supports hiPSC attachment, spreading, and differentiation; but also allows for long-term hiPSC self-renewal in a defined medium. We demonstrated that hiPSCs retained stable proliferation and pluripotency markers after growth on the Synthemax Surface for ten consecutive passages. Our experimental results suggest that Synthemax Surface in combination with defined medium can provide a defined culture system for expansion of hiPSC for cell therapy applications.
