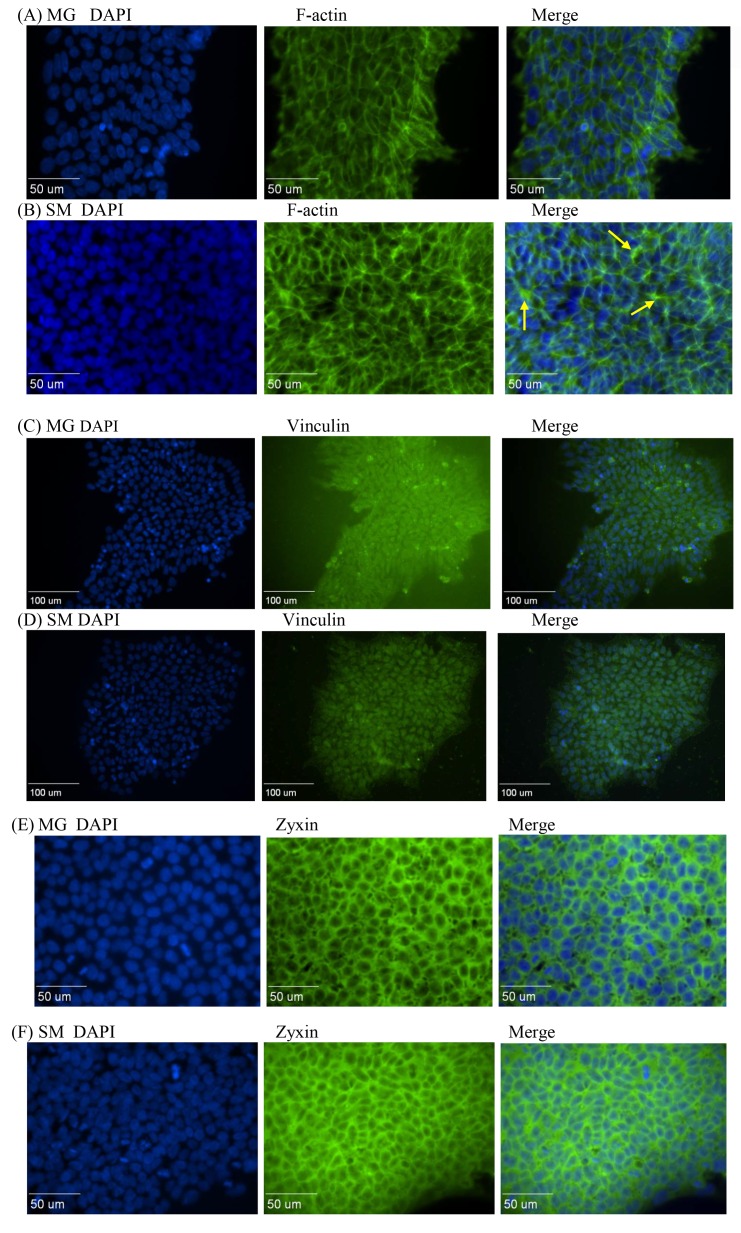# Correction: A Synthetic, Xeno-Free Peptide Surface for Expansion and Directed Differentiation of Human Induced Pluripotent Stem Cells

**DOI:** 10.1371/annotation/0c321570-b9b8-44eb-9368-f174720d0d61

**Published:** 2013-02-27

**Authors:** Sha Jin, Huantong Yao, Jennifer L. Weber, Zara K. Melkoumian, Kaiming Ye

Portions of Figures 1 and 4 were omitted. The full versions can be viewed here:

Fig 1: 

**Figure:**
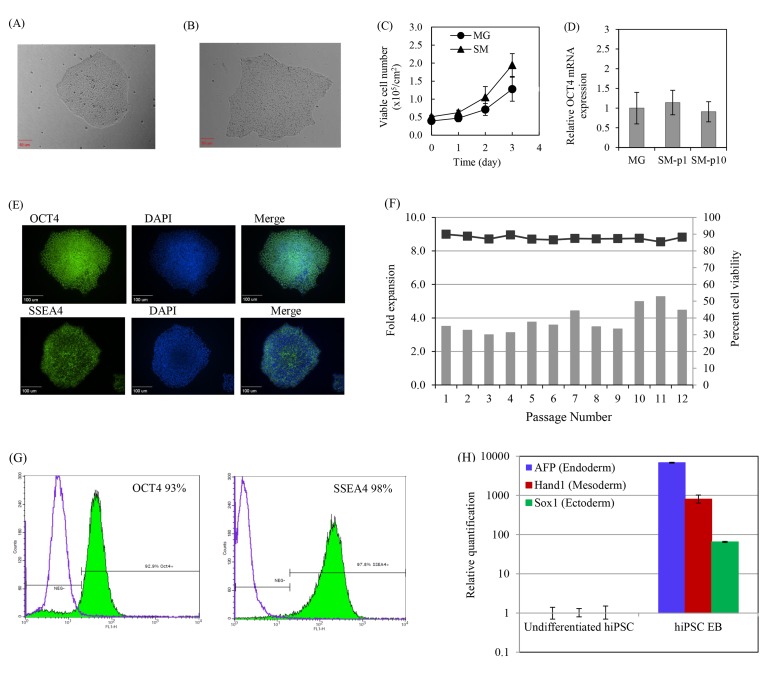


Fig 4: 

**Figure:**